# Cross-Linked Enzyme Aggregates of Naringinase: Novel Biocatalysts for Naringin Hydrolysis

**DOI:** 10.4061/2011/851272

**Published:** 2011-09-06

**Authors:** Maria H. L. Ribeiro, Marco Rabaça

**Affiliations:** Research Institute for Medicines and Pharmaceutical Sciences (i-Med-UL), Faculty of Pharmacy, University of Lisbon, Av. Prof. Gama Pinto, 1649-003 Lisbon, Portugal

## Abstract

Cross-linked enzyme aggregates (CLEAs) have emerged as interesting biocatalyst design for immobilization. These new generation enzyme biocatalysts, CLEAs, in addition to exhibiting good mechanical stability, can be highly active, since they do not include large amounts of foreign particulate nonenzymatic material and may have increased stability. Naringinase (NGase) is an enzyme complex with high potential in pharmaceutical and food industries. In fact, NGase can be used in the biotransformation of steroids, of antibiotics and mainly on glycosides hydrolysis. In this paper, the formation of CLEAs was tried using ammonium sulphate, polyethylene glycol 6000 and *tert*-butyl alcohol as precipitant agents and glutaraldehyde as cross-linking agent, at different pH, time, and temperature conditions. However, among the precipitant agents tested, only *tert*-butyl alcohol cross-linked with glutaraldehyde allowed the formation of CLEAs, at pH 4.0 and at temperature between 7 and 10°C. Different enzyme loadings were tested. The NGase-CLEAs were highly effective in naringin hydrolysis. The operational stability of the NGase-CLEAs aggregates was studied through six successive reutilizations.

## 1. Introduction

Cross-linked enzyme aggregates (CLEAs) have emerged as interesting biocatalysts design for immobilization. Additionally, since precipitation can be used on the purification of enzymes, and on the preparation of CLEAs, this technique may integrate in a single/or reduced operation: enzyme purification and immobilization.

This new generation of biocatalysts, CLEAs, in addition to exhibiting good mechanical stability, can be highly active, since they do not include large amounts of foreign particulate nonenzymatic material and may have increased stability. The CLEAs technology present many advantages on different applications, as it is simple and amenable to rapid optimization, leading to low costs and short time-to-market processes. The self-immobilization techniques of CLEAs are referred to as providing higher volumetric and specific activity.

The cross-linking agents such as glutaraldehyde, ethylene polymers and aldehyde dextran, bind to the enzymes without the need for support when they are close [1–3].

Immobilization by cross-linked enzyme aggregates (CLEAs) involves the precipitation mechanism of enzymes by salting out, addition of nonionic polymers or mixture of solvents. The most common used precipitating agents are ammonium sulphate, polyethylene glycol (PEG 6000, PEG 8000), or *ter*-butyl alcohol among others [3, 4].

Therefore, the formation of CLEAS comprises two distinct steps. The first involves the physical aggregation of the enzyme by nondenaturing methods, to further establish the chemical bonds between the enzymes by cross-linked via cross-linking agents [1, 3–5]. Precipitation, by the addition of salts, organic solvents, or nonionic polymers to aqueous solutions of proteins, is a commonly used method for protein purification [1]. The resulting physical aggregates of enzyme molecules are supramolecular structures that are held together by noncovalent bonding and can be easily redissolved in water. The formation of CLEAs is based on the reaction between glutaraldehyde or other cross-linking agent and the NH_2_ groups located on the surface of the enzyme, leading to formation of insoluble aggregates, stable and catalytically active [2–6]. Due to the different biochemical and structural properties of proteins the best precipitant and cross-linker can vary from one enzyme to another.

The nature of the enzyme, the residues of serine and lysine mainly present in the enzyme protein, the number of protein subunits are among the many factors that influence the formation of CLEAs [1, 5, 6]. In addition to the properties of enzymes, the mode and duration of the precipitation process, the concentration of cross-linking agent, and the conditions of temperature and pH are other factors with great importance [1, 5].

This method of immobilization can lead to changes in enzyme activity due to the establishment of chemical bonds that can alter the enzyme structure and lead to a loss or increase in activity [1, 5]. These modifications may be used to model the selectivity of the enzyme for a particular substrate or product formation, or lead to a change in the main use of the enzyme [1, 2, 5]. In fact, it is not unusual to have a modification of enzyme activity and selectivity when resorting to the formation of CLEAs, which may lead to different applicant with additional benefit in the stabilization of enzymes, including their three-dimensional structure [1, 2, 4, 5].

Therefore, the technology of CLEAs an alternative immobilization procedure has several advantages in the industrial context and appears as a simple, economical, and easy optimization for the immobilization of enzymes, allowing the selection and retention of enzymatic activity in aqueous media and within a wide range of pH and temperature [5, 7, 8].

Naringinase is an enzyme complex obtained from fungi such as *Penicillium decumbens*, *Aspergillus* sp. *Aspergillus niger*, *Aspergillus oryzae,* or *Rhizopus nigricans*. It is a heterodimer of 168 kDa composed of two subunits, the *α*-L-ramnosidase and *β*-D-glucosidase [9, 10]. The hydrolysis of naringin into prunin liberates one molecule of rhamnose while the hydrolysis of prunin into naringenin liberates one molecule of glucose.

The high cost of naringinase production has limited its industrial application. To overcome this problem, the cloning and expression of the gene for *α*-L-rhamnosidase in *E. coli* are very promising [11], increasing the potential applications in pharmaceutical and food industries [9, 12–15]. Naringinase can be used in the biotransformation of steroids, antibiotics, and mainly on glycosides hydrolysis. Among flavonoids, naringin can be hydrolyzed by naringinase to naringenin and to the reducing sugars ramnose and glucose. Naringin, the substrate used in this bioconversion, and the product, its aglycone, naringenin, are compounds with important biological and pharmacological activities, such as anti-oxidant, anti-inflammatory, or anticancer. Simultaneously, naringinase enables sweeten fruit juices, as naringin has a bitter taste and naringenin is tasteless, maintaining product stability, organoleptic characteristics, and other inherent properties of antioxidant compounds, leading to increased business value [9, 10, 16].

The purpose of this work was the development and optimization of biocatalysts using the immobilization in cross-link aggregates (CLEAs). This work, using as model system the hydrolysis of naringin to naringenin and reducing sugars with naringinase encapsulated in CLEAs, aims to provide a suitable approach on selecting a suitable set of precipitant, cross-linking and polymerization agents, and conditions to enzyme immobilization.

## 2. Materials and Methods

### 2.1. Enzyme and Chemicals

Naringinase (CAS no. 9068-31-9, cat. No. 1385) from *Penicillium decumbens *was obtained from Sigma-Aldrich and stored at −20°C. The lyophilized naringinase (NGase) was dissolved in the appropriate buffer solution and kept at 4°C until use. Naringin and naringenin were obtained from Sigma-Aldrich, St. Louis, Mo, USA. All other reagents used were of analytical grade and obtained from different sources.

### 2.2. Analytical Methods

The reducing sugars concentration was determined using the dinitrosalicylic acid (DNS) microassay at *λ* = 545 nm (*BioPhotometer *spectrophotometer) [17].

The protein content was determined using Bradford microassay, from Bio-Rad protein procedure, at *λ* = 595 nm (*BioPhotometer *spectrophotometer), using a naringinase calibration curve [18].

### 2.3. Preparation of CLEAs

The formation of NGase-CLEAs was tried using the precipitant agents, ammonium sulphate, polyethylene glycol 6000 and alcohol *tert*-butyl and glutaraldehyde as cross-linking agent, at different pH, time, naringinase concentration, and temperature conditions. 

Initially, the preparation of CLEAs was carried out in 24 microwell plates, adding 0.5 mL of naringinase (0.5 mg/mL) to 0.5, 1.0, 1.5, and 2.0 mL of ammonium sulphate 50% (w/v) and sodium acetate buffer (pH = 4.0) to a final volume of 2.5 mL. After 2, 6, 24, and 48 h were added 0.5 mL of glutaraldehyde 25% (v/v) to each well of the microtiter plate.

PEG 6000 was another precipitant tested. This study was carried out adding 2.0 mL of naringinase solutions with different concentrations (0.5, 1.0, 1.5, and 2.0 mg/mL), in acetate buffer pH = 4.0, to PEG 6000 and stirred at 60 rpm for 1 hour. Then, to make the cross-linking occur, 0.5 mL of glutaraldehyde 25% (v/v) was added and kept stirring for 2 h. CLEAs formation occurred during 48 h. After that, they were centrifuged at 5000 rpm for 10 min. The supernatant was removed and the CLEAs were suspended in acetate buffer 20 mM pH 4.0, centrifuged at 5000 rpm for 10 minutes. This step was repeated twice and the CLEAs used in bioconversion trials.

The immobilization of naringinase was also performed using *tert*-butyl alcohol as precipitant agent. 2 mL of naringinase solutions with different concentrations (0.5, 1.0, 1.5, 2.0, 4.0, 6.0, and 8.0 mg/mL) in acetate buffer 20 mM, pH = 4.0, was placed in each tube, added with 2.0 mL of *tert*-butyl alcohol and left at 60 rpm for 1 hour.

The cross-linking reaction was performed by adding 0.3 mL and 0.5 mL of glutaraldehyde 25% (v/v) and proceeded, under shaking at 60 rpm for 2 h. The formation of CLEAs was left to occur for 48 h. Afterwards, they were centrifuged at 5000 rpm for 20 min. The supernatant was removed and NGase-CLEAs were suspended in acetate buffer solution. Centrifuged again at 5000 rpm for 20 minutes, this step was repeated twice. 

The above mentioned procedure was repeated with naringinase solutions of different concentrations at pH 6.0 and pH 10.0.

### 2.4. Bioconversion Studies

The naringin hydrolysis was carried out in standard solutions in acetate buffer (20.0 mM), at pH 4.0. The naringin concentration varied from 0.5 to 4.0 mg/mL. A naringinase concentration of 500 mg/L was used in free and immobilized enzyme studies. 

The CLEAs aggregate activities were evaluated following the reducing sugars formation by DNS method and the protein in reaction media was evaluated following Bradford's method.

After collecting samples, the reaction was immediately stopped by lowering the temperature below 0°C. The enzyme activity was evaluated according to the initial rate method, through determination of reducing sugars concentration of the collected samples, using DNS assay. Each data point of kinetic measurements was performed in an individual experiment, in triplicate. 

The fit of the Michaelis-Menten model {*v* = ([*S*] · *v*_max_)/([S] + *k*_*M*_)}{*v*—initial rate (mM min^−1^)), [*S*]—substrate concentration (mM), *v*_max _-maximum initial rate (mM min^−1^), *k*_*M*_-Michaelis-Menten constant (mM)} to experimental data was carried out through nonlinear regression by minimising the residual sum of squares between the experimental data points of the initial rate versus substrate concentration and those estimated by the model, using Solver add-in from Microsoft Excel 2003 for Windows XP, considering the following options: Newton method; 100 iterations, precision of 10^−6^, 5% of tolerance and 1 × 10^−4^ convergence. The nonlinear regression parameters were constricted to positive numbers.

### 2.5. Stability Studies

To test the stability of the immobilized naringinase, the NGase-CLEAs were reused six times on the hydrolysis of naringin, in acetate buffer 20 mM, pH 4.0 (Figure 2). After each run of 60 minutes, the biocatalyst were separated and washed with acetate buffer 20 mM, pH 4.0. The reaction medium was then replaced by fresh medium and a new run took place.

### 2.6. Effect of Temperature and Substrate Concentration

The effect of substrate concentration and temperature on the hydrolysis of naringin by naringinase encapsulated on CLEAs was studied according to response surface methodology (RSM) based on a central composite rotational design (CCRD). The selection of the values of two variables under study for the experimental design was developed, based on the results of preliminary tests, using the computer program “StatisticaTM” (version 6 from Statsoft, USA). This program allowed the calculation of values for the sets of experimental points of the CCRD design from the central point that has been established. The five levels coded and decoded to the point set of experimental variables, temperature, and concentration of naringin are shown in Table 1. 

The reaction was promoted by adding NGase-CLEAs to 2 mL of sodium acetate buffer (20 mM, pH = 4.0) and 2 mL of naringin solutions with concentrations equal to those expressed in the table on the design experimental CCRD (Table 1). The reaction occurs for 30 minutes at different temperatures specified in Table 1. Aliquots of 75 *μ*L at the end of the experiments were withdrawn, to carry out the determination of reducing sugars by DNS method. 

## 3. Results and Discussion

Initially, the immobilization of naringinase in cross-linked aggregates (NGase-CLEAs) was tried using ammonium sulphate as precipitation agent and different naringinase concentrations. However, there was any formation of precipitate and even after 48 hours at 0°C, no modification was observed in the reaction medium. Also, the addition of the cross-linking agent, glutaraldehyde, to the different naringinase solutions (1 mg/mL to 4 mg/mL) did not result in aggregate formation. Nevertheless, it led to the appearance of a yellow to brown color, depending on the exposure time (2, 6, 24, 48 h). This fact suggests the occurrence of a colorimetric reaction between glutaraldehyde and ammonium sulphate and/or proteins, without precipitation. 

Therefore, others precipitant agents were tested for NGase-CLEAs formation, namely, polyethylene glycol 6000 (PEG 6000) and *tert*-butyl alcohol. With the precipitant, PEG 6000, an increase in viscosity was observed, after agitation of the medium for 1 h. The addition of the cross-linking agent, glutaraldehyde, led to the formation of enzyme aggregates, which increased with storage for 48 hours in an ice bath. Afterwards, these PEG-NGase-CLEAs were used in naringin bioconversion trials. However, these PEG-NGase-CLEAs were unstable in sodium acetate buffer, leading to a suspension, even after successive centrifugations. 

**Figure 1:**
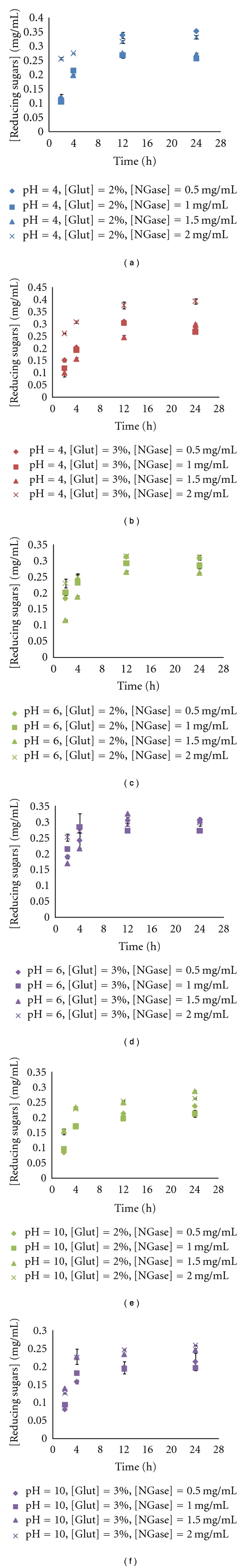
Reducing sugars formation with NGase-CLEAs with [Naringin] = 0.5 mg/mL, pH 4.0, temperature 30°C, and 100 rpm. The CLEAs were produced with the precipitant *tert*-butyl alcohol, at pH 4.0, 6.0 and 10.0, with different naringinase concentrations and the cross-linking agent, glutaraldehyde at 2% and 3%.

The precipitant agent, *tert*-butyl alcohol, was studied in naringinase solutions at different pH values (4.0, 6.0, and 10.0) (Figure 1), following the studies of Sheldon [1] and Wilson et al. [4] that mentioned the need of higher pH to form CLEAs. In addition, the influence of naringinase concentration and glutaraldehyde was also evaluated (Figure 1). 

The addition of tert-butyl alcohol to different environments at temperatures of 7°C and 10°C led to crystallization of the alcohol. This problem was overcome with agitation at 60 rpm for 1 h, allowing the dissolution and the formation of a solution with tert-butyl alcohol [50% (v/v)] and naringinase solutions. However, these conditions did not allow the formation of a precipitate, unlike observed by Sheldon [1] and Wilson et al. [4] with penicillin acylase. Therefore, only the addition of the cross-linking agent, glutaraldehyde, under shaking led to the formation of a suspension. This suspension, after 12 h to 24 h, had a lattice aspect and 48 h later consistent aggregates were formed, after successive centrifugations. 

The increase of pH, respectively, from 4.0 to 6.0 and 10.0 resulted in smaller amounts of aggregates formation. Moreover, in order to reduce the time for CLEAs formation, bicarbonate buffer and borohydride sodium were added to the medium containing naringinase, tert-butyl alcohol, and glutaraldehyde. However, after 2 h, there was no CLEAs formation. This result was the opposite to what had been described by Cao et al. [3] on the immobilization of penicillin acylase.

In this work, an increased formation of cross-linked aggregates was attained with higher enzyme concentrations (2–4 mg/mL), despite the amount of glutaraldehyde added (2 and 3%). This improvement may be attributed to the amino acids involved in bonding between the enzyme and glutaraldehyde. Some authors reported that the number of lysine residues present in the enzyme [5, 6] is an important factor in the formation of stables CLEAs, like the ratio of protein/glutaraldhyde [3]. 

In further work, the NGase-CLEAs were obtained with the precipitant, tert-butyl alcohol, and the cross-linking agent, glutaraldehyde during 48 h.

### 3.1. NGase-CLEAs Activity Studies

The naringin hydrolysis was carried out with naringinase immobilized on CLEAs (*tert*-butyl alcohol plus glutaraldehyde), at 30°C, under shaking of 100 rpm. This hydrolysis was carried out using NGase-CLEAs produced at various pH (4.0, 6.0, and 10.0), with different naringinase concentrations (0.5, 1.0, 2.0, 4.0 mg/mL) for 24 h (Figure 1). 

A decreased in reducing sugars concentration was observed in CLEAs produced with naringinase immobilized at higher pH (6.0 and 10.0) (Figure 1). 

At pH 4.0 using glutaraldehyde in a concentration of 2% and 3%, and naringinase in a concentration of 0.5 mg/mL and 2.0 mg/mL led, respectively, to the formation of 0.34 mg/mL and 0.39 mg/mL of reducing sugars (Figure 1). Noteworthy was the high initial activity of naringinase in NGase-CLEAs, namely after 2 h, a reducing sugars concentration of 0.261 mg/mL was obtained.

Further studies were performed with NGase-CLEAs, pH 4.0, *tert*-butyl alcohol, and glutaraldehyde (3%), namely, time-course, substrate concentration and stability.

A linear relation for reducing sugars formation was observed during the first 30 minutes enzymatic reaction, in reaction time-course (10, 20, 30, 60, and 120 minutes) evaluation with different naringin concentration, at 30°C, 100 rpm.

**Figure 2:**
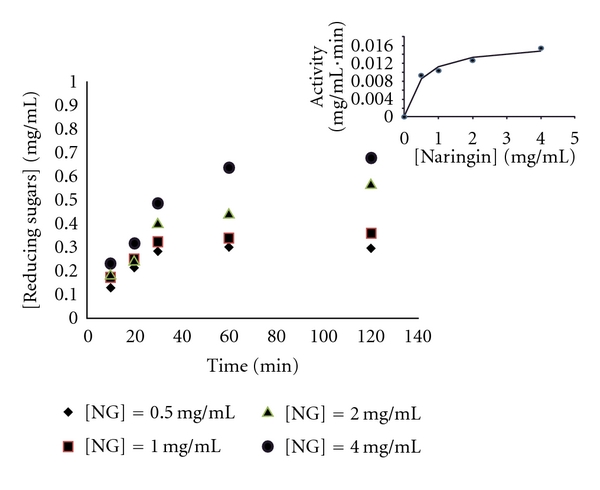
Time-course of reducing sugars formation and naringinase activity using NGase-CLEAs (precipitant: tert-butyl alcohol and cross-linking agent: glutaraldehyde 3%) in acetate buffer (20 mM) pH 4.0, with different naringin concentrations, at 30°C, 100 rpm.

An activity of 20 *μ*g/min·mL was obtained with naringinase in a concentration of 0.5 mg/mL (Figure 2). I.A.C. Ribeiro and M.H.L. Ribeiro [19] described enzymatic activities of 12 *μ*g/mL·min with naringinase immobilized in k-carrageenan, at 30°C, and pH 4.0.

Different naringin concentrations were hydrolyzed to rhamnose and glucose and naringenin, allowing kinetic constants evaluation (Figure 2). The kinetic profile of naringin hydrolysis using immobilized naringinase in CLEAs obeys to the Michaelis–Menten model. The values of *K*_*m*_ and *V*_max_ were, respectively, 0.36 mM and 0.127 mM/min. Vila Real et al. [9] referred a *K*_*m*_ of 0.55 mM and a *V*_max_ of 0.063 mM/min for free naringinase at 30°C and pH 4.0, while Pedro et al. [20] mentioned values of 0.303 mM and 0.042 mM/min for naringinase immobilized in calcium alginate.

### 3.2. Operational Stability of NGase-CLEAs

The production of an immobilized form of a biocatalyst is typically performed in a batch run, where large amounts result. It is therefore a key issue, particularly if large scale application is envisaged, to ensure that the decay of catalytic activity of the immobilized biocatalyst is as reduced as possible. Since such decay is nevertheless inevitable, it is considered to be of relevance to have a sound estimate of the rate of such decay, irrespectively of the pattern of stability decay profile of the free enzyme.

**Figure 3:**
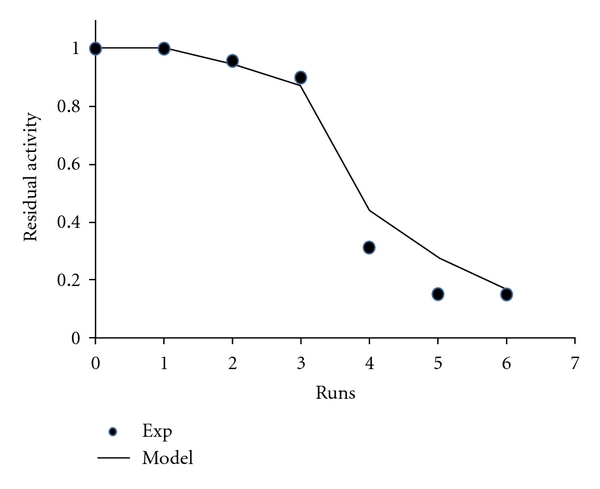
Operational stability of naringinase (NGase-CLEAs), at 30°C, acetate buffer pH 4.0, 100 rpm.

In this work, the operational stability of NGase-CLEAs was evaluated at 30°C, pH 4.0, 100 rpm with naringin in a concentration of 0.5 mg/mL. The activity of the freshly prepared matrices in the first run was defined as 100% (*A*_0_). The naringinase residual activity was evaluated and as shown in Figure 3, above 90% until the third use. A decrease to 30% was observed after four reutilizations. 

To describe the deactivation kinetics, each experimental run was converted to the fraction of the original activity, that is, its residual activity. This residual activity, *A*_*r*_, (*A* × *A*_0_^−1^), was defined as the ratio between the specific activity after each run (*A*) and the specific activity of the first run (*A*_0_). 

The reuse of the encapsulated naringinase can be described adequately by a serial enzyme deactivation sequence (1) involving a first-order deactivation sequence and an active intermediate.

(1) $$A_{r}=\left[1+\frac{\alpha_{1}k_{1}}{k_{2}-k_{1}}\right]e^{-k_{1}t}-\left[\frac{\alpha_{1}k_{1}}{k_{2}-k_{1}}\right]e^{{-k}_{2}t},$$

*k*_1_ and *k*_2_ are first and second deactivation rate coefficients, respectively; *A*_0_, *A*_1_, and *A*_2_ are specific activities of the initial active enzyme, enzyme intermediate, and final enzyme state, respectively; *α*_1_ and *α*_2_ are the specific activities ratio *A*_1_ × *A*_0_^−1^ and *A*_2_ × *A*_0_^−1^, respectively [21]. The fit of Sadana's model (1) to experimental data was carried out using “Solver” add-in from Excel for Windows, version 8.0 SR2, by minimising the residual sum of squares between the experimental data points and those estimated by the respective model and considering the following options: Newton method; 100 iterations, precision of 10^−5^; 5% of tolerance and 0.001 convergence. 

A biphasic deactivation nature was observed, where the final state was totally inactivated (*α*_2_ = 0). A *k*_1_ of 1.26 h^−1^ and an *α*_1_ of 1.76 were obtained.

The half-life of the biocatalyst, *t*1/2, that is, the operation time required for half the enzyme activity to be lost as a result of deactivation was of 4.5 h. 

### 3.3. Modelling NGase-CLEAs Activity as a Function of Substrate and Temperature

Naringin hydrolysis was performed using naringinase encapsulated on CLEAs, according to a statistical design “central composite rotatable design” (CCRD), depending on the concentration of naringin and temperature. The experiments were carried out according to a factorial design 2^2^ and a CCRD, as a function of both naringin concentration (NG) and temperature (*T*).

**Figure 4:**
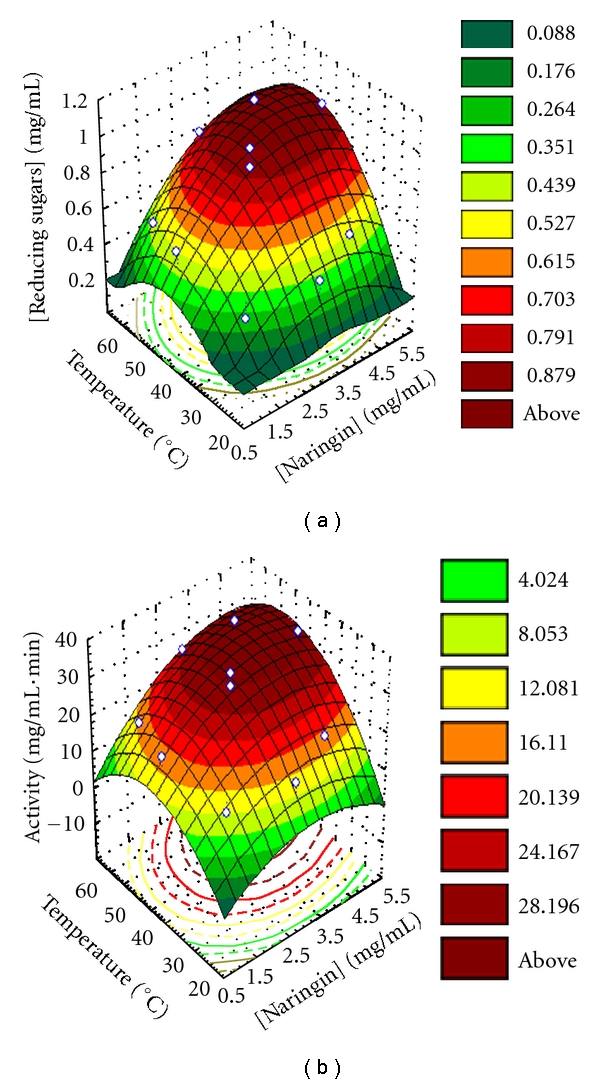
Response surface fitted to the experimental data points, corresponding to naringinase residual activity, as function of naringin concentration and temperature, with NGase-CLEAs, in acetate buffer (20 mM) pH 4.0.

The experimental results showed that reducing sugars formation and naringinase activity was affected by temperature and naringin concentration individually and interactively (Figure 4). 

The significant effects of naringin concentration and temperature and interaction of (Naringin) × Temperature on the reducing formation and naringinase activity are shown in Table 2. 

A least-squares technique was used to fit quadratic polynomial models and obtain multiple regression coefficients for reducing sugars formation and naringinase activity which are summarized in Table 3. Examination of these coefficients indicated that linear and quadratic terms of naringin concentration and temperature, respectively, were significant *P* < 0.01 for reducing sugars formation and naringinase activity (Table 2). A positive interaction between the variables tested (*T* × NG) on naringinase activity indicated that higher activities are obtained at higher temperatures and naringin concentration. 

Therefore, curved surfaces were fitted to the experimental data (Figure 4). Partial differentiation of these polynomial equations was used to find the optimum points, that is, the stationary points. The least-square estimates of the coefficients of the model were calculated from the values of the response for each experiment in the chosen experimental matrix. The relationships between independent and dependent variables in the three-dimensional representations are convex surfaces, for reducing sugars formation and naringinase activity (Figure 4). The obtained response surface (Figure 4) was described by second-order polynomial equations to the experimental data points, as a function of temperature and naringin concentration (Table 3). In the design of these models, the significant effects (*P* < 0.05), and those that presented a confident range smaller than the value of the effect or smaller than the standard deviation were included in these model equations. In fact, these later effects have a lower probability, but their values are not small enough to be neglected.

The high values of *R* ^2^ and *R*_*adj*_^2^ of the model (Table 3) showed a close agreement between the experimental results and the theoretical values predicted by the model [22]. The adjusted coefficients of determination for reducing sugars formation and naringinase activity (*R*_*adj*_^2^ = 0.815) implied that 81.5% of the variations could be explained by the fitted model. 

Enzyme activity appears to be optimal within the ranges tested. A concentration of reducing sugars of 1.0 mg/mL and a maximum naringinase activity of 30 mg/mL·min is expected at 50°C with 3 mg/mL of naringin. 

Once tested, the model may be used to predict the value of the response(s) under any conditions within the experimental region. 

In future work, this system will be tested in addition to naringinase immobilization, to enzyme purification.

## 4. Conclusions

In this work, NGase-CLEAs were produced with a high enzymatic activity, allowing the hydrolysis of naringin. 

NGase-CLEAs with greater stability and a higher activity of 20 *μ*g/mL·min were produced with *tert*-butyl alcohol as precipitating agent, glutaraldehyde (3%) as cross-linking agent, at pH 4, and temperature between 7°C and 10°C. 

The response surface methodology (RSM) enabled the assessment of the combined effects of temperature and naringin concentration for reducing sugars production and naringinase activity. The modeling of hydrolysis of naringin, with temperature and naringin concentration, catalyzed by NGase-CLEAs was described by response surfaces of convex form, and a maximum naringinase activity of 30 mg/mL·min is expected at 50°C with 3 mg/mL of naringin.

## Figures and Tables

**Table 1:** Coded and decoded level for experimental variables, naringin concentration, and temperature.

CCRD	[Naringin] (mg/L)	Temperature (°C)
−*√*2	0.9	23.8
−1	1.5	30.0
0	3.0	45.0
+1	4.5	60.0
+*√*2	5.1	66.2

**Table 2:** Effects and respective significance levels (*p*) of [Naringin] and temperature (*T*) on reducing sugars formation and naringinase residual activity (*A*_*r*_).

Factor	[Reducing sugars] (mg/mL)		Activity (mg/mL·min)	
		(*p*)		(*p*)
[Naringin] (linear)	0.3471	0.0094	11.578	0.0094
[Naringin] (quadratic)	−0.2340	0.0666	−7.804	0.0665
Temperature (linear)	0.2381	0.0475	7.944	0.0477
Temperature (quadratic)	−0.4551	0.0107	−15.175	0.0107
[Naringin] × temperature	0.1555	0.2491	5.175	0.2497

*Naringin hydrolysis in acetate buffer (20 mM) pH 4.0 with NGase-CLEAs (NGase 2 mg/mL).

**Table 3:** Second-order model equations for the response surfaces fitted to the experimental data points of reducing sugars formation and naringinase residual activity (*A*_*r*_) against naringin (NG) and temperature (*T*).

System	Model equations	*R* ^2^	*R*_*adj*_^2^
NGase-CLEAs	[Reducing sugars] = −1.887 + 0.272 [NG] − 0.052 [NG]^2^ + 0.089 *T* − 0.001 *T* ^2^ + 0.0035 [NG] × *T*	0.910	0.815
	[Activity] = −62.959 + 2.955 [NG] − 0.034 [NG]^2^ + 9.089 *T* − 1.734 *T* ^2^ + 0.115 [NG] × *T *	0.910	0.815
